# A Novel Method for the Description of Voltage-Gated Ionic Currents Based on Action Potential Clamp Results—Application to Hippocampal Mossy Fiber Boutons

**DOI:** 10.3389/fncel.2015.00514

**Published:** 2016-01-13

**Authors:** John R. Clay

**Affiliations:** Department of Physiology, National Institute of Neurological Disorders and Stroke, National Institutes of HealthRockville, MD, USA

**Keywords:** rat hippocampus, action potential clamp, mathematical models, Goldman-Hodgkin-Katz, ion channels

## Abstract

Action potential clamp (AP-clamp) recordings of the delayed rectifier K^+^ current *I*_K_ and the fast-activated Na^+^ current *I*_Na_ in rat hippocampal mossy fiber boutons (MFBs) are analyzed using a computational technique recently reported. The method is implemented using a digitized AP from an MFB and computationally applying that data set to published models of *I*_K_ and *I*_Na_. These numerical results are compared with experimental AP-clamp recordings. The *I*_Na_ result is consistent with experiment; the *I*_*K*_ result is not. The difficulty with the *I*_K_ model concerns the fully activated current-voltage relation, which is described here by the Goldman-Hodgkin-Katz dependence on the driving force (*V*-*E*_K_) rather than (*V*-*E*_K_) itself, the standard model for this aspect of ion permeation. That revision leads to the second—a much steeper voltage dependent activation curve for *I*_K_ than the one obtained from normalization of a family of *I*_K_ records by (*V*-*E*_K_). The revised model provides an improved description of the AP-clamp measurement of *I*_K_ in MFBs compared with the standard approach. The method described here is general. It can be used to test models of ionic currents in any excitable cell. In this way it provides a novel approach to the relationship between ionic current and membrane excitability in neurons.

## Introduction

The action potential clamp technique (AP-clamp) is a paradigm in which an AP recorded from a neuron in current clamp is applied to that same cell in voltage clamp mode before and after the addition of a specific ion channel blocker to the external medium (Llinás et al., 1982; Bean, 2007). In this way the role of that current during an AP can be determined. The AP can also be applied computationally to a mathematical model of that current constructed from voltage step results in order to provide an additional test of the model. This approach was recently applied to *I*_Na_and *I*_Ca_ in suprachiasmatic nucleus neurons (Jackson et al., 2004; Clay, 2015). In this report the method is applied to the AP-clamp recordings of Alle et al. (2009) of *I*_Na_ and *I*_K_ in rat hippocampal mossy fiber boutons (MFBs) at physiological temperatures (*T* = 36−37°C). Those results demonstrate a significant separation in time during an AP of *I*_Na_ and *I*_K_, an important result for efficient neuronal signaling (Crotty et al., 2006; Alle et al., 2009; Sengupta et al., 2010). The method was implemented using digitized results from MFBs (personal communication, Dr. H. Alle). A digitized representation of an AP from an MFB was applied computationally to the models of *I*_K_ and *I*_Na_ in MFBs of Engel and Jonas (2005) for model testing. The *I*_K_ AP-clamp analysis revealed a significant discrepancy between theory and experiment, which can be resolved using the Goldman-Hodgkin-Katz (GHK) equation for the fully activated current-voltage relation for *I*_K_ (Clay, 2009). The revised *I*_*K*_ model provides a significant improvement in the description of this component compared with a model in which a linear dependence of *I*_K_ on (*V*-*E*_K_) was used. Computational analysis of the *I*_Na_ AP-clamp result (Alle et al., 2009) using the Engel and Jonas (2005) *I*_Na_ model was in agreement with experiment.

## Materials and methods

A data set corresponding to an AP from MFBs was applied computationally to the models of *I*_K_ and *I*_Na_ of Engel and Jonas (2005) which are given by *I*_K_ = *g*_K_*n*^4^(*V*-*E*_K_) and *I*_Na_ = *g*_Na_*m*^3^*h*(*V*-*E*_Na_), respectively, similar to the original models of *I*_K_ and *I*_Na_ in squid giant axons of Hodgkin and Huxley (1952), with *g*_K_ and *g*_Na_constants, *E*_K_ and *E*_Na_ the K^+^, and Na^+^ reversal potentials and

(1) $$\begin{array}{r} \text{d}x∕\text{d}t=-\left(\alpha_{x}+\beta_{x}\right)x+\alpha_{x} \end{array}$$

with *x* = *n, m*, or *h*, and time *t* in msec. The rate parameters in Equation (1) are given by Engel and Jonas (2005).

(2) $$\begin{array}{rcl} \alpha_{n} & = & -0.01\left(V+55\right)∕\left\{\text{exp}\left[-\left(V+55\right)∕10\right]-1\right\};\\ \beta_{\text{n}} & = & 0.125\text{ exp}\left[-\left(V+65\right)∕80\right] \end{array}$$

(3) $$\begin{array}{rcl} \alpha_{m} & = & -93.8\left(V-105\right)∕\left\{\text{exp}\left[-\left(V-105\right)∕17.7\right)-1\right\};\\ \beta_{m} & = & 0.17\text{ exp}\left(-V∕23.3\right)\\ \alpha_{h} & = & 0.00035\text{ exp}\left(-V∕18.7\right);\\ \beta_{h} & = & 6.6∕\left\{\text{exp}\left[-\left(V+17.7\right)∕13.3\right]+1\right\}. \end{array}$$

The expressions for α_*n*_ and β_*n*_ (Equation 2) were taken from the experimental procedures of Engel and Jonas (2005). The expressions for α_*m*_, β_*m*_, α_*h*_, and β_*h*_ (Equation 3) were taken from Supplementary Table 1 of their paper. All αs and βs in Equations (2) and (3) are in units of inverse milliseconds. The model of *I*_K_ was based on the voltage step recordings of this component in MFBs by Geiger and Jonas (2000) obtained at *T* = 34°C. It was extrapolated to *T* = 37°C using a Q_10_ of 2.2 (Fohlmeister, 2015). That is, α_*n*_ and β_*n*_ (Equation 2) were each multiplied by 1.27. The reversal potential for K^+^ used in the analysis was *E*_K_ = −110 mV. [*E*_K_ = *kT/q* ln(${\text{K}}_{\text{o}}^{+}$/${\text{K}}_{\text{i}}^{+}$) where *k* is the Boltzmann constant, *T* is the absolute temperature, *q* is the unit electron charge (*kT*/*q* = 26.7 mV for *T* = 37°C), ${\text{K}}_{\text{o}}^{+}$=2.5 mM and ${\text{K}}_{\text{i}}^{+}$ = 155 mM (Alle et al., 2009)]. The recordings of *I*_Na_ of Engel and Jonas (2005) were obtained at *T* = 23°C. Their model of *I*_*Na*_ was extrapolated to *T* = 37°by multiplying each of the αs and βs in Equation (3) by a factor of 2.8. The reversal potential for Na^+^ was *E*_Na_ = 62 mV (Alle et al., 2009).

**Figure 1:**
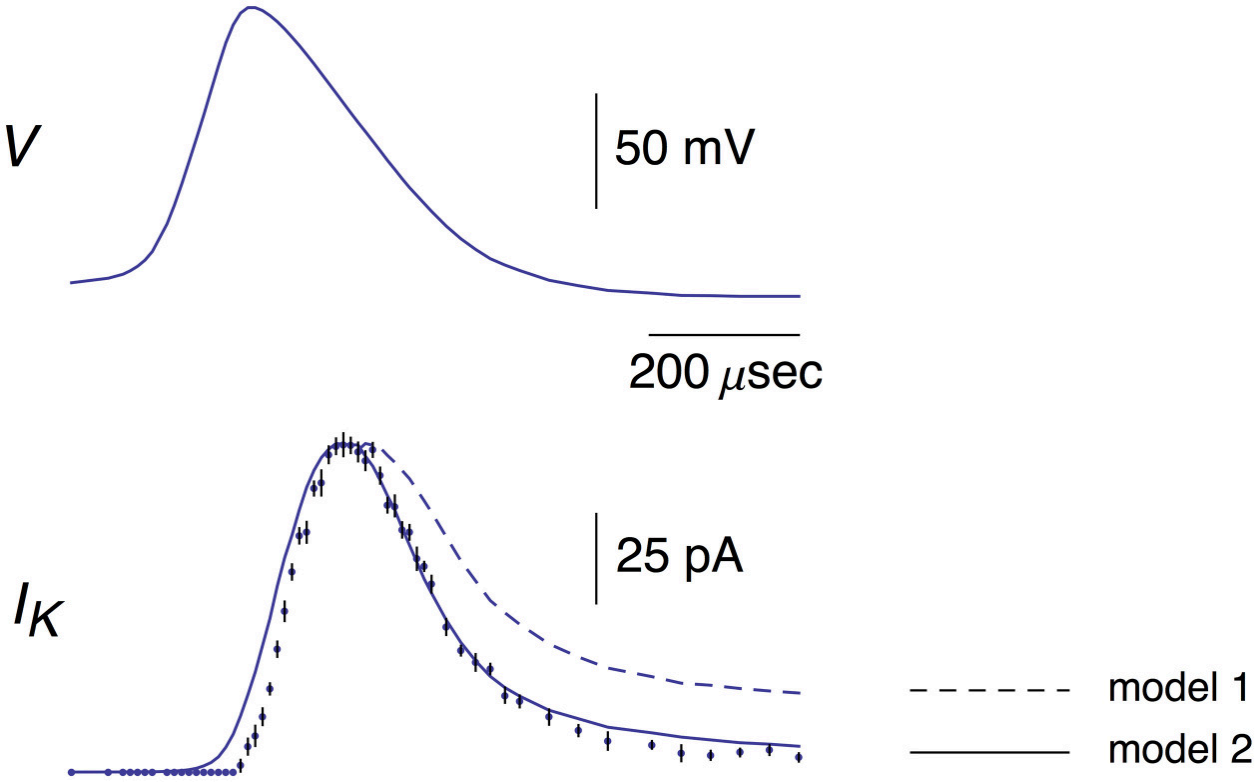
**Top panel:** This AP corresponds to the AP in the top panel of Figure 1B from Alle et al. (2009). A digitized version of this waveform is given in Supplementary Table 1. The points in that data set were connected by lines to given the result shown. **Bottom panel**: The data points with error bars (*n* = 9; ±SEM) correspond to AP-clamp measurements of *I*_K_ in MFBs (Alle et al., 2009). The mean values of these results are also given in Supplementary Table 1. The theoretical results (models 1 and 2) are as described in the text.

The *V*_*i*_ vs. *t*_*i*_ data set (*i* = 1,2,3…  ; Supplementary Table 1) of the MFB AP from Figure 1B of Alle et al. (2009) is represented in the top panel of Figure 1 with lines connecting adjacent points. It was applied to Equation (1) with *x* = *n, m*, or *h* using NDSolve in Mathematica (Wolfram Research, Inc., Champaign, IL). At *t*_1_ = 0, *V*_1_ = −80 mV. The initial value of *n, n*_1_, was given by α_*n*_/(α_*n*_+β_*n*_) with *V* = −80 mV and α_*n*_and β_*n*_ given by Equation (2), i.e., *n*_1_ = 0.1288. The following point of the AP data set is *t*_2_ = 0.039 ms, *V*_2_ = −77.7 mV. The corresponding value of *n*, *n*_2_, was determined from Equation (1) and NDSolve using *V*(*t*) = *V*_1_+(*V*_2_−*V*_1_)(*t*−*t*_1_)/(*t*_2_−*t*_1_) for *t*_1_< *t*< *t*_2_. The result was *n*_2_ = 0.1289. More significant changes in *n* occur later as the membrane potential is depolarized throughout the AP. A similar analysis was applied to *x* = *m* and *h*.

## Results

### K^+^ current

**Figure 2:**
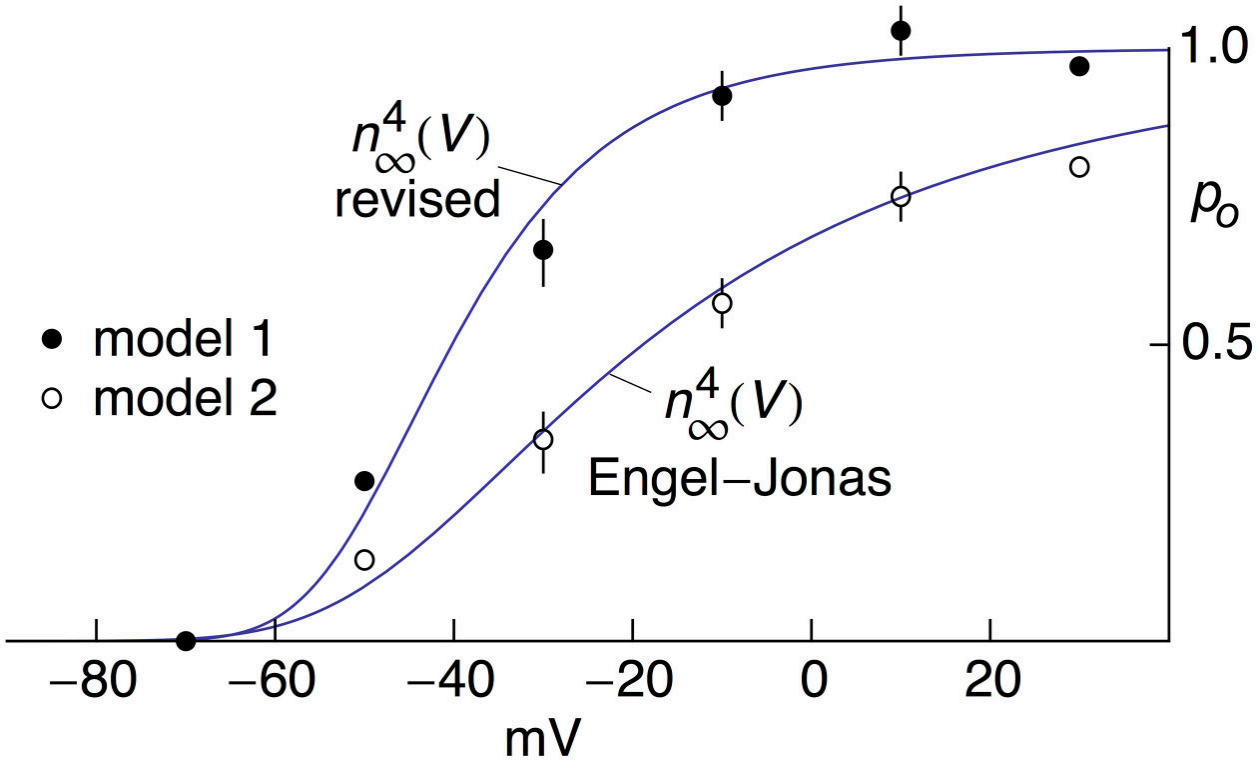
**Open channel probability, ***p***_***o***_, for ***I***_**K**_ in MFB's as a function of ***V*****. The open circles were taken from Figure 5C from Geiger and Jonas (2000). The curve describing those results corresponds to $n_{\infty }^{4}$(*V*) with *n*_∞_(*V*) = α_*n*_∕(α_*n*_+β_*n*_) and α_*n*_ = −0.01(*V*+55)/{exp[−(*V*+55)/10]−1} msec^−1^, β_*n*_ = 0.125 exp[−(*V*+65)/80] msec^−1^ (Engel and Jonas, 2005). These results were obtained from normalization of a family of *I*_K_ records with (*V*-*E*_K_) and *E*_K_ = −85 mV (Geiger and Jonas, 2000). They were multiplied by (*V*+85) and renormalized using GHK[(*V*−*E*_K_)] as described in the text with *kT*/*q*=26.5 mV (*T* = 34°C) and *E*_K_ = −104 mV from K_*o*_ = 2.5 mM and K_*i*_ = 125 mM (Geiger and Jonas, 2000). The renormalized results are represented by the filled circles. The theoretical curve describing those results is given by $n_{\infty }^{4}$(*V*) with α_*n*_= −0.01(*V*+55)/{exp[−(*V*+55)/10]−1} msec^−1^ and β_*n*_ = 0.125 exp[−(*V*+65)/20] msec ^−1^.

A digital representation of the AP-clamp recordings of *I*_K_ from Alle et al. (2009) is given in the bottom panel of Figure 1 of this report (data points with the error bars representing ±SEM, *n* = 9; Supplementary Table 1). These results are the differences obtained by application of the AP in Figure 1 to MFBs in voltage-clamp before and after addition to the bath of 1 mM 4-aminopyridine (4-AP), which was sufficient to completely block *I*_K_ elicited by an AP (Alle et al., 2009, 2011). They were scaled to match the *I*_*K*_ result in the bottom panel of Figure 1B of Alle et al. (2009). Also shown in Figure 1 is the prediction of the Engel and Jonas (2005) *I*_K_ model (dashed line; model 1) starting from the maximum level of *I*_K_ close to the peak of the AP. The rising phase of the model is not shown. These results (also given in Supplementary Table 1) correspond to *I*_K_,_*i*_ = *g*_K_*n*_*i*_^4^(*V*_*i*_-*E*_K_), *i* = 1.2.3… , with *n*_*i*_ determined as described above (Section Materials and Methods), *E*_K_ = −110 mV, and *g*_*K*_ = 36 mS/cm^2^. This model—model 1, the Engel and Jonas (2005) *I*_K_ model—does not provide a good description of the *I*_K_ AP-clamp result. The difficulty most likely concerns the fully activated current-voltage relation for *I*_K_. This result for squid axons is well described by the GHK dependence on (*V*-*E*_K_) rather than by (*V*-*E*_K_) itself (Clay et al., 2008), a relationship given by *I*_K_(*n* = 1) = *a*GHK[(*V*-*E*_K_)] where *a* is a constant, and GHK[(*V*-*E*_K_)] = (*qV*/*kT*) {exp[*q*(*V*-*E*_K_)/*kT*]-1}/[exp(*qV*/*kT*)-1]. In their original analysis of squid axon currents Hodgkin and Huxley (1952) obtained the *I*_K_ activation curve, an important result for models of *I*_K_, by normalizing a family of *I*_K_ records with (*V*-*E*_K_), a linear dependence on driving force. Normalization by GHK[(*V*-*E*_K_)] should be used instead. This analysis is illustrated for *I*_K_ from MFBs in Figure 2 using the results of Geiger and Jonas (2000). Their *I*_K_activation curve is shown in Figure 2 (open circles) along with a description of this result by $n_{\infty }^{4}$(*V*) with *n*_∞_(*V*) = α_*n*_/(α_*n*_+β_*n*_) and α_*n*_ and β_*n*_ as given above (Equation 2). Their result for *V* = 50 mV (Figure 1C of Geiger and Jonas, 2000) was not included. The *I*_K_ component in squid axons is partially blocked by ${\text{Na}}_{i}^{+}$ in a voltage-dependent manner for strong depolarizations such as *V* ≥ 50 mV (Bezanilla and Armstrong, 1972; French and Wells, 1977). Geiger and Jonas (2000) used an intracellular solution containing 21 mM Na^+^. A partial block of *I*_*K*_ in MFBs at 50 mV by this level of ${\text{Na}}_{i}^{+}$ cannot be ruled out and so this point was excluded from the analysis. The remaining results from *V* = −70 to +30 mV were multiplied by (*V*-*E*_K_) to remove the linear normalization they used to obtain their result. The next step was normalization with GHK[(*V*-*E*_K_)] as described in Clay (2009)—closed circles in Figure 2. The result is an *I*_K_ activation curve that is significantly steeper than the one obtained using normalization by (*V*-*E*_K_). A single modification in the Engel and Jonas (2005) model is sufficient to describe these results, namely a change in β_*n*_ from 0.125 exp[−(*V*+65)/80] ms^−1^ to 0.125 exp[−(*V*+65)/20] ms^−1^, the curve labeled “$n_{\infty }^{4}$ revised” in Figure 2. The same modification in the original Hodgkin and Huxley (1952) model, namely replacing “80” in the exponential term of β_*n*_ to “20,” is sufficient to describe the *I*_K_ activation curve in squid axons obtained using GHK normalization of a family of *I*_K_ records (Clay et al., 2008). The AP-clamp result for this version of the Engel and Jonas (2005) *I*_K_ model—model 2—is given by *an*_*i*_^4^ GHK[(*V*_*i*_-*E*_K_)], continuous line in Figure 1, with *a* = 1.3 mA/cm^2^ and *n*_*i*_ determined from Equation (1) using the modified version of β_*n*_. This result provides a significant improvement in the description of the falling phase of the experimental record compared with model 1. The rising phase of both models underestimates the delay in the rise of *I*_K_ during an AP, a result that is similar to the Cole and Moore (1960) effect for voltage steps in squid axons (Discussion).

**Figure 3:**
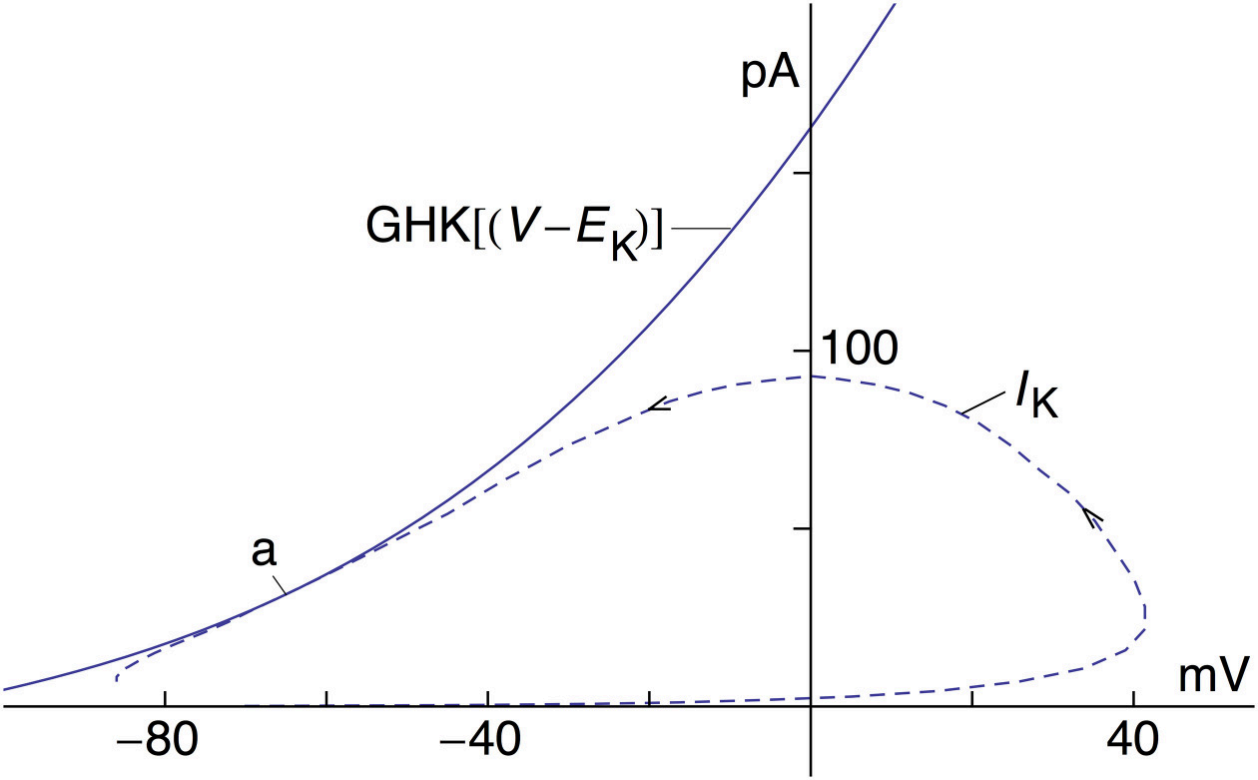
**Current-voltage trajectory (dashed line) of ***I***_**K**_—model 2—for the AP in Figure 1**. The arrows indicate the direction of time. Also shown is the GHK current-voltage relation, *an*^4^ GHK(*V*-*E*_K_) with *a* = 1.3 mA/cm^2^, *n* = 0.283, and GHK(*V*-*E*_K_) as given in the text. The *I*_K_ trajectory during the AP is tangent to the GHK relation at point **a**.

The revised *I*_K_ result in Figure 1—model 2—is further illustrated by the current-voltage trajectory for *I*_K_ during the AP in Figure 1 (Figure 3—dashed line). The *I*_K_ gate—the *n* variable—is maximally activated during the AP to a level of 0.283, which occurs near the latter part of the repolarization phase. The GHK current voltage relation with *n* = 0.283, also shown in Figure 3, is tangent to the trajectory at this point (arrow labeled **a**). The trajectory lies close to the GHK relation a considerable distance on either side of **a**, indicating that the time course of *I*_K_ in model 2 during repolarization is largely determined by the GHK equation.

### Na^+^ current

**Figure 4:**
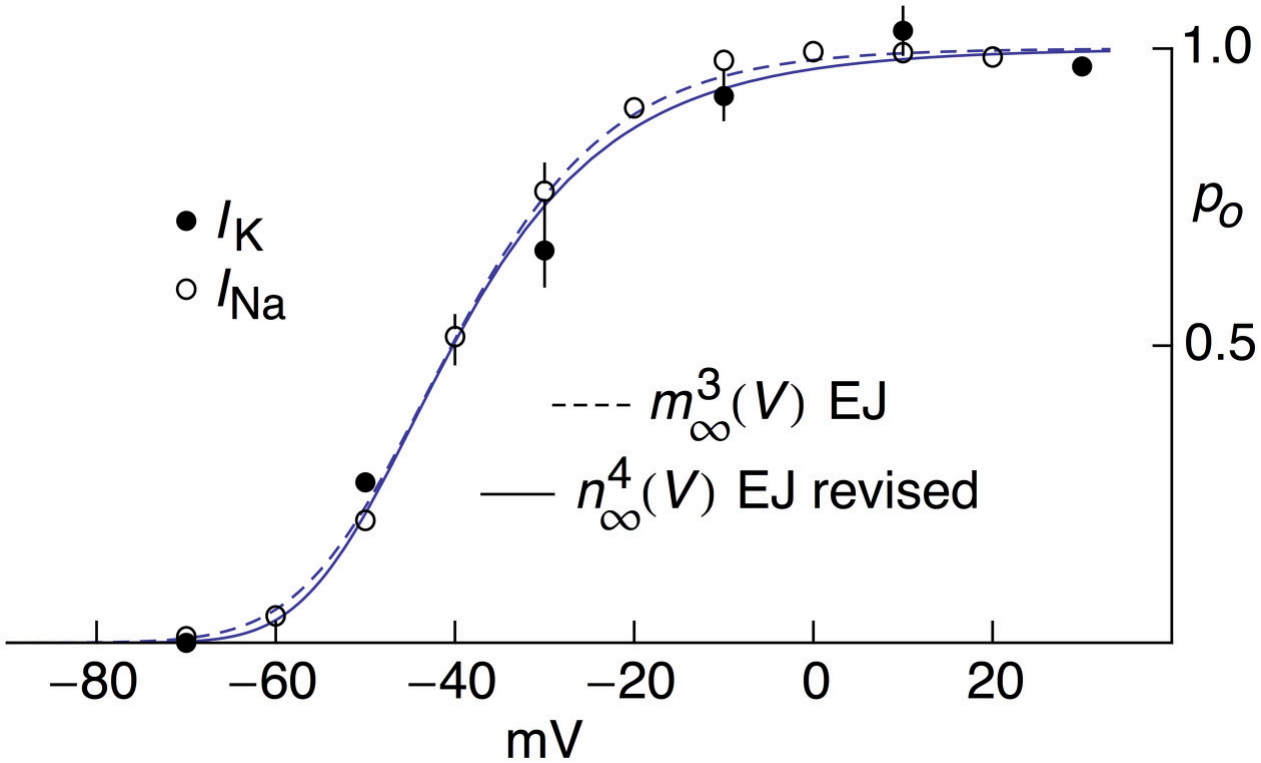
**Open channel probability, ***p***_***o***_, for ***I***_**K**_ and ***I***_**Na**_ for MFBs as a function of ***V***, both experimental and theoretical**. The experimental results for *I*_K_ were taken from Figure 5C of Geiger and Jonas (2000) as modified in Figure 2. The solid line labeled “*n*^4∞^(*V*) EJ revised” corresponds to [α_*n*_/(α_*n*_+β_*n*_)]^4^ with α_*n*_ = −0.01(*V*+55)/{exp[−(*V*+55)∕10]−1} msec^−1^ and β_*n*_ = 0.125 exp[−(*V*+65)∕20] msec ^−1^. The experimental results for *I*_Na_ were taken from Figure 2B of Engel and Jonas (2005). The dashed curve labeled “*m*^3∞^(*V*) EJ” corresponds to [α_*m*_/(α_*m*_+β_*m*_)]^3^ with α_*m*_ = −93.8(*V*−105)/{exp[−(*V*−105)∕17.7]−1} msec^−1^ and β_*m*_ = 0.17 exp(−*V*∕23.3) msec^−1^.

Activation curve results for *I*_Na_ from MFBs—both experimental and theoretical—taken from Engel and Jonas (2005) are shown in Figure 4 along with the revised *I*_K_ results described above. The *I*_K_ and *I*_Na_ activation curves overlap almost completely (Figure 4) an observation that may be consistent with known structural similarities of voltage-gated Na^+^ and K^+^ channels (MacKinnon, 1991, 1995; Jan and Jan, 1997; Hanlon and Wallace, 2002).

**Figure 5:**
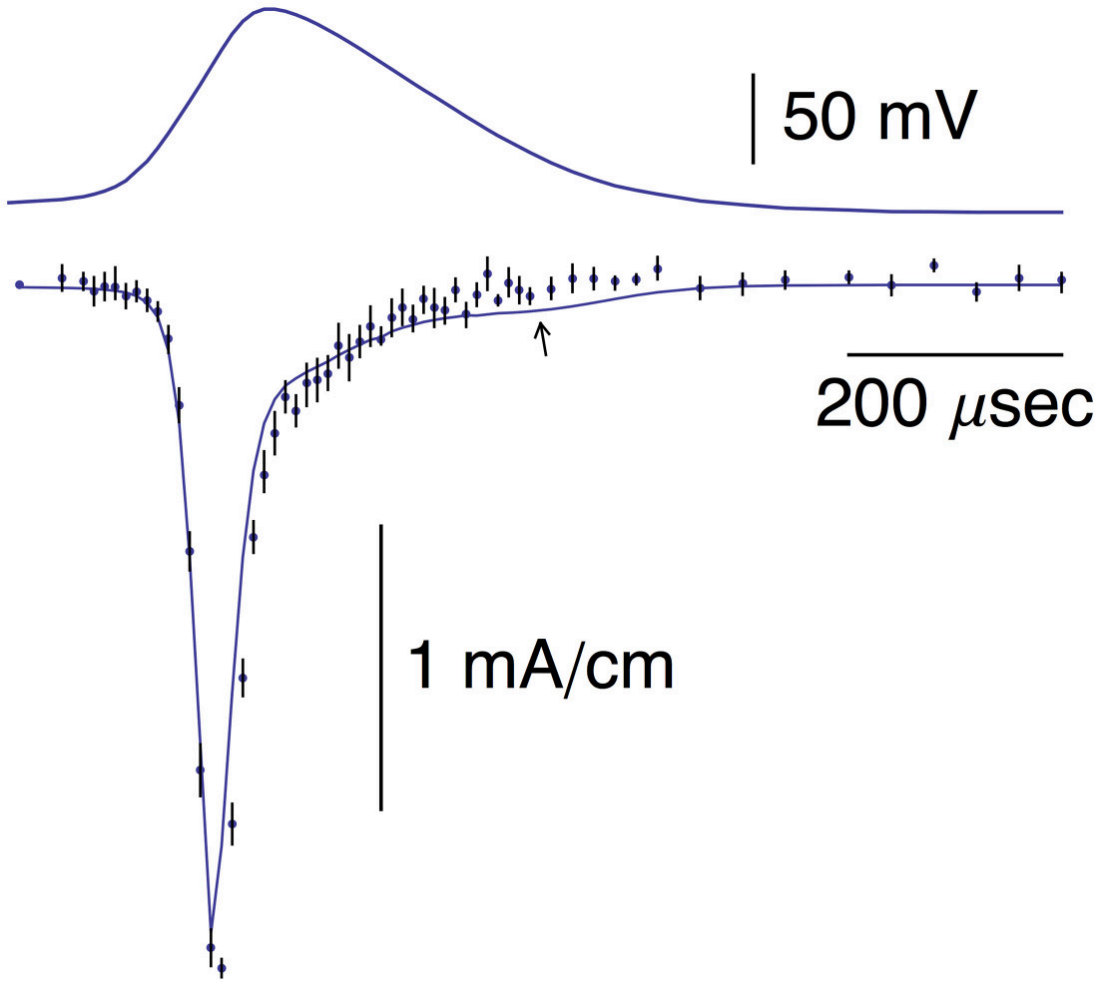
**Top panel:** Same AP as in the top panel of Figure 1. **Bottom panel:** The data points with error bars (*n* = 8; ±SEM) correspond to AP-clamp measurements of *I*_Na_ in MFBs (Alle et al., 2009). The mean values of those results are also given in Supplementary Table 1. The theoretical curve is as described in the text.

Pooled results of AP-clamp recordings of *I*_Na_ from Alle et al. (2009) are illustrated in Figure 5 (data points with error bars representing ±SEM, *n* = 9; Supplementary Table 1). They were scaled to match the *I*_Na_ result in the bottom panel of Figure 2B of Alle et al. (2009). The results are the differences obtained by applying the AP shown in the top panel of Figure 5 to MFBs before and after the addition of 1 μM tetrodotoxin (TTX) to the bathing medium. Also shown in Figure 5 is the prediction of the Engel and Jonas (2005) model described above, *I*_Na_,_*i*_ = *g*_Na_*m*_*i*_^3^*h*_*i*_(*V*_*i*_-*E*_Na_), with *i* = 1.2,3…, *g*_Na_ = 110 mS/cm^2^, *m*_*i*_ and *h*_*i*_ determined as described in Section Materials and Methods and *E*_Na_ = 62 mV (Alle et al., 2009). The model provides a good description of the experimental results. The arrow in Figure 5 highlights a slight secondary increase of *I*_Na_ during repolarization in the Engel and Jonas (2005) *I*_Na_ model attributable to an overlap of activation and inactivation. A similar result is not apparent in the experimental recordings.

**Figure 6:**
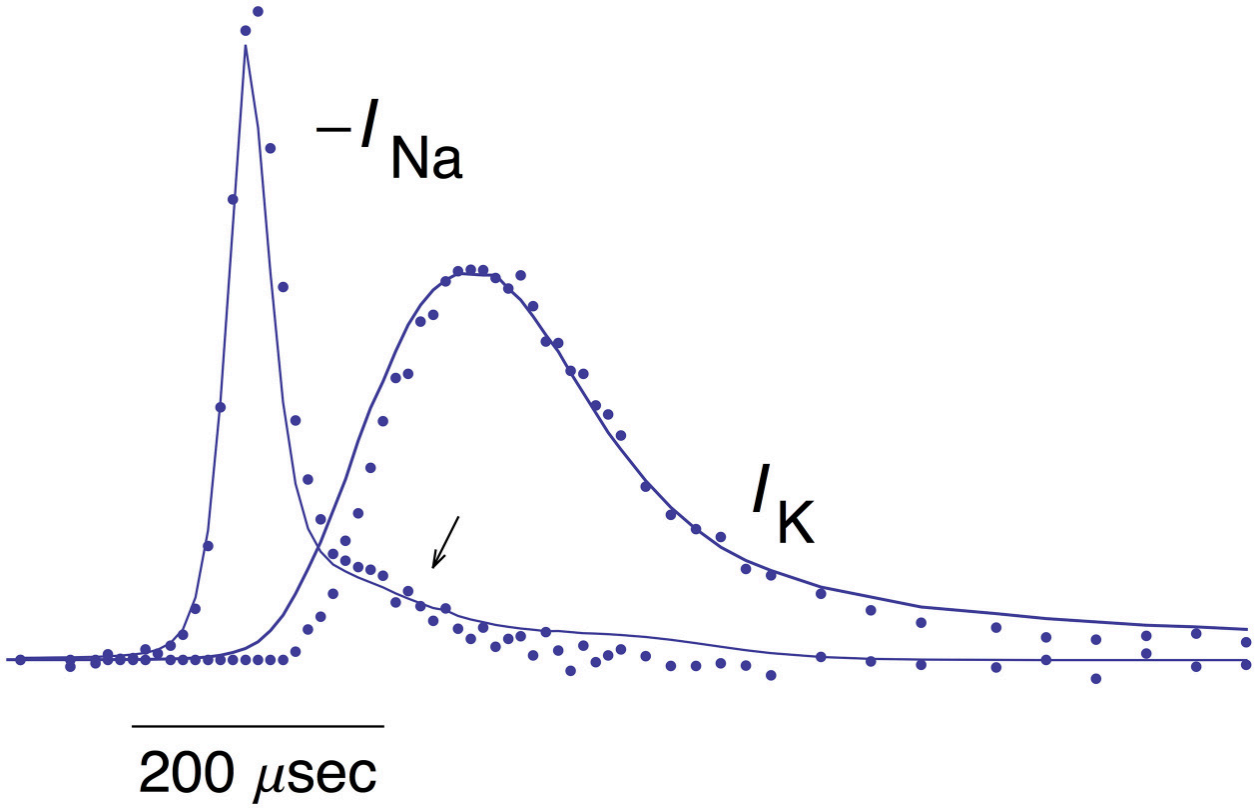
**Pooled results for –***I***_**Na**_ and ***I***_**K**_ from MFBs**. The mean values of these measurements are shown here along with the theoretical curves for –*I*_Na_ and *I*_K_ are the same as described in Figures 1, 5, respectively. The arrow highlights a slight overlap in time of the two results (Alle et al., 2009).

The mean of the pooled results for *I*_K_ and –*I*_Na_ from Figures 1, 5, respectively, are shown in Figure 6 scaled as described in Alle et al. (2009) along with the predictions of the *I*_Na_ model and *I*_K_ model 2 described above. The arrow in Figure 6 highlights a slight overlap of *I*_Na_ with *I*_K_during the AP, an energetically inefficient result (Alle et al., 2009).

## Discussion

This report provides further illustration of a method recently described for the analysis of ionic currents recorded with the AP-clamp technique (Clay, 2015). The work also provides an example of the utility of the GHK equation for the analysis of *I*_K_ from a mammalian neuronal preparation. Traditionally, those results have been described by *I*_K_ = *g*_K_(*V*-*E*_K_) with *g*_K_ a constant (Hodgkin and Huxley, 1952). This expression implies, by definition, that the slope conductance for *I*_K_ at a given potential below *E*_K_ is the same as the slope conductance positive to *E*_K_. This result is theoretically impossible when *E*_K_ ≠ 0 because *I*_K_ is proportional to ${\text{K}}_{o}^{+}$ with *V* well below *E*_K_ and *I*_K_ is proportional to ${\text{K}}_{i}^{+}$ when *V* is well above *E*_K_. The fully activated current-voltage relation for *I*_K_ outwardly rectifies, a result that is well described by the GHK equation (Clay, 2009). A similar result applies for *I*_Na_ with a caveat. The fully activated current-voltage relation for *I*_Na_ in squid axons in Ca^2+^-free seawater is consistent with the GHK equation (Vandenberg and Bezanilla, 1991; their Figure 3). It inwardly rectifies since ${\text{Na}}_{o}^{+}$ is much greater than ${\text{Na}}_{i}^{+}$. Calcium ions in normal seawater partially block *I*_Na_ in a voltage-dependent manner with the blockade increasing as the membrane potential is hyperpolarized relative to *E*_Na_. This effect counterbalances the inward rectification of *I*_Na_ in the absence of divalent cations so that *I*_Na_ is approximately proportional to (*V*-*E*_Na_) for physiological conditions over the range of potentials spanned by an AP (Vandenberg and Bezanilla, 1991). A similar mechanism may apply to *I*_*Na*_ from other preparations (Worley et al., 1986; Green et al., 1987).

One consequence of the original prediction by Hodgkin and Huxley (1952) that *I*_K_ = *g*_K_(*V*-*E*_K_) is that activation curves for voltage gated K^+^ channels have typically been determined by normalizing a family of *I*_K_ records using (*V*-*E*_K_). An activation curve with a shallow voltage dependence is obtained (open circles, Figure 2). In contrast, normalization of those results by GHK[(*V*-*E*_K_)] yields an activation curve having a steepness similar to that noted by Sigworth (2003) for voltage-gated K^+^ channels. Moreover, the revised K^+^ channel activation curve is similar to the Na^+^ channel activation curve (Figure 4). High sensitivity of these channels to voltage is important because cellular voltage changes are small (Sigworth, 2003).

Models 1 and 2 for *I*_K_ both fail to account for the delay in the rising phase of this component during an AP (Figures 1, 6), a result that is similar to the Cole and Moore (1960) effect in squid axons. Specifically, the delay in the rising phase of *I*_*K*_ following a voltage clamp step from relatively negative holding potentials is greater than the prediction of the Hodgkin and Huxley (1952) *n*^4^ model (Cole and Moore, 1960). This result is significant in squid axons even for moderately hyperpolarized holding potentials such as −75 mV (Figure 5; Clay et al., 2008). The discrepancy between theory and experiment reported here for the rising phase of *I*_*K*_ elicited during AP-clamp from a holding potential of −80 mV in MFBs is a corollary of the Cole and Moore (1960) effect.

The *I*_K_ component underlying repolarization in rat hippocampal MFBs is the result of the entry of K^+^ through a mixture of channels, Kv1, Kv3, and BK (Alle et al., 2011). BK channels appear not to be significant for basal APs, i.e., APs recorded under normal physiological conditions (Alle et al., 2011). The model of *I*_K_ in MFB's by Engel and Jonas (2005) is based, implicitly, on the assumption of a homogeneous population of K^+^ channels. They noted that their model provided “a relatively accurate description of the voltage-dependence of activation of K^+^ channels in MFBs.” This view is not necessarily at odds with the results of Alle et al. (2011) especially with regard to Kv, channels that are activated rapidly. The kinetics of Kv1 and Kv3 may well be described by the same, or similar, Hodgkin and Huxley (1952) type model. In any event the falling phase of *I*_K_ obtained in AP-clamp from MFBs is consistent with a homogeneous population of K^+^ channels with their fully-activated current-voltage relation described by GHK(*V*-*E*_K_).

The emphasis in this report is on a method, for analyzing ionic currents in neurons with an application to MFBs. The method is general. It can be applied to ionic currents in any excitable cell for which a specific blocker is available, such as TTX for *I*_Na_. The method requires a digitized representation of an experimentally recorded AP as well as a model of the ionic current in question obtained from voltage clamp step results such as the Hodgkin and Huxley (1952) *m*^3^*h* model for *I*_Na_. The analysis given above for *I*_Na_ in MFBs is largely confirmatory of the *m*^3^*h* model as given by Engel and Jonas (2005). The analysis for *I*_K_ in MFBs reveals two discrepancies between experiment and the Hodgkin and Huxley (1952) model of *I*_K_, one concerning the rising phase of *I*_K_ during an AP similar to the Cole and Moore (1960) effect and a discrepancy in the falling phase that can be accounted for by changing the fully-activated current-voltage for *I*_K_ from a linear dependence upon the K^+^ driving force to the GHK dependence on (*V*-*E*_K_). The method provides a complementary test of models constructed from voltage step results. An AP-clamp rapidly scans the range of membrane potentials corresponding to this waveform. In this way the GHK dependence of *I*_K_ on (*V*-*E*_K_) can be elucidated for the physiological range of membrane potentials more readily than is possible with voltage steps.

The original work of Hodgkin and Huxley (1952) continues to influence the design and analysis of experimentals in membrane neuroscience. The method described in this report provides a variation of their approach that can yield additional insight to the relationship between membrane excitability and the ionic currents that underlie excitability.

### Conflict of interest statement

The author declares that the research was conducted in the absence of any commercial or financial relationships that could be construed as a potential conflict of interest.
